# Supplementary material for: Novel ratio-metric features enable the identification of new driver genes across cancer types
Source: Sci Rep. 2022 Jan 7;12:5. doi: 10.1038/s41598-021-04015-y (PMC8741763; doi:10.1038/s41598-021-04015-y)
Supplement: Supplementary file 1 — Supplementary Figures. [file 41598_2021_4015_MOESM1_ESM.pdf]

Supplementary Material

Supplementary Figures

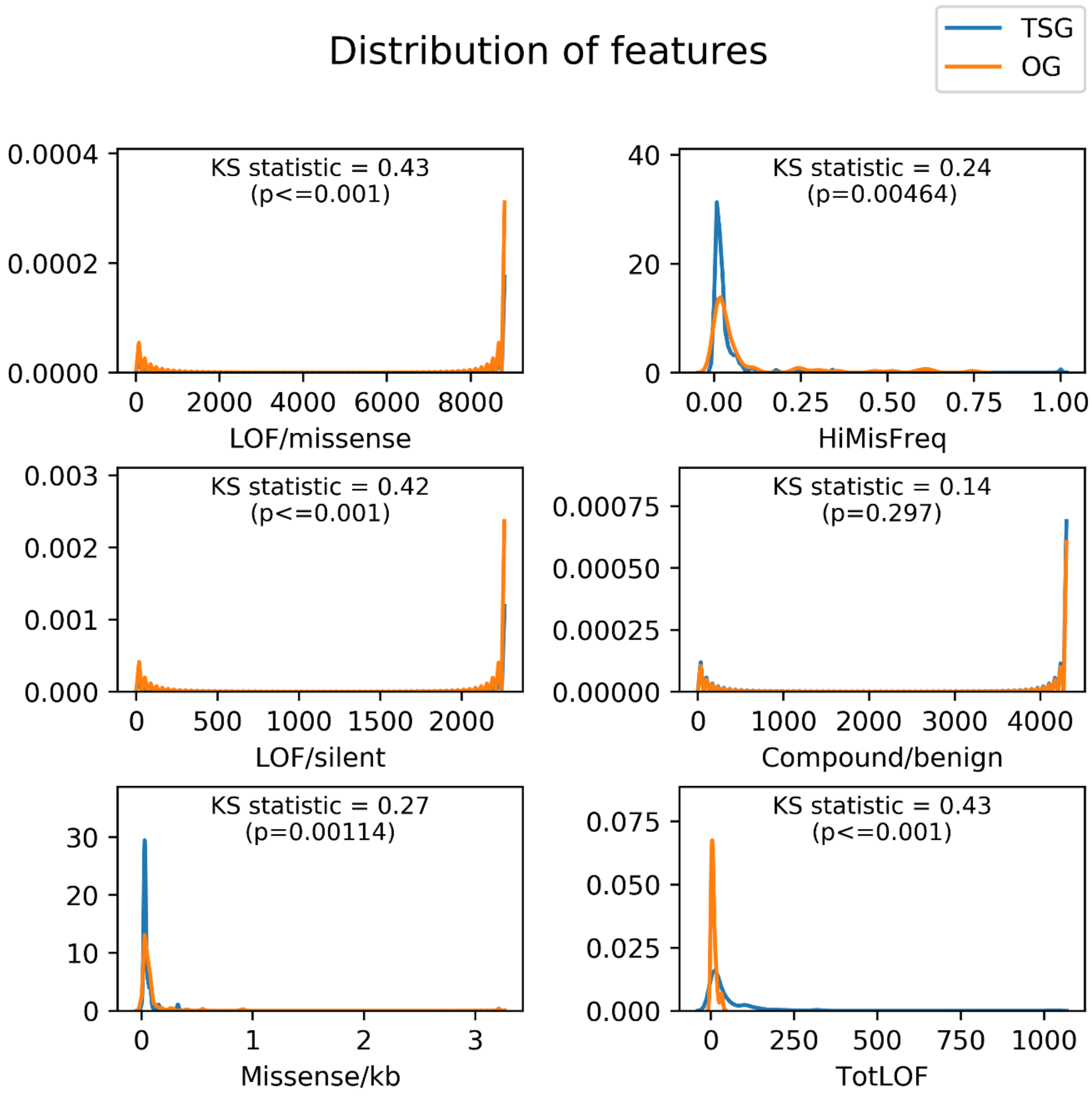

Supplementary Figure 1a Distribution of features used by the classifier for TSG and OG. The kernel density was plotted for training genes used for classifying TSG and OG to study the differences between the distributions of features. Kolmogorov-Smirnov statistic and the p-value is given for each feature. Larger KS statistic means a larger difference in the two distributions.

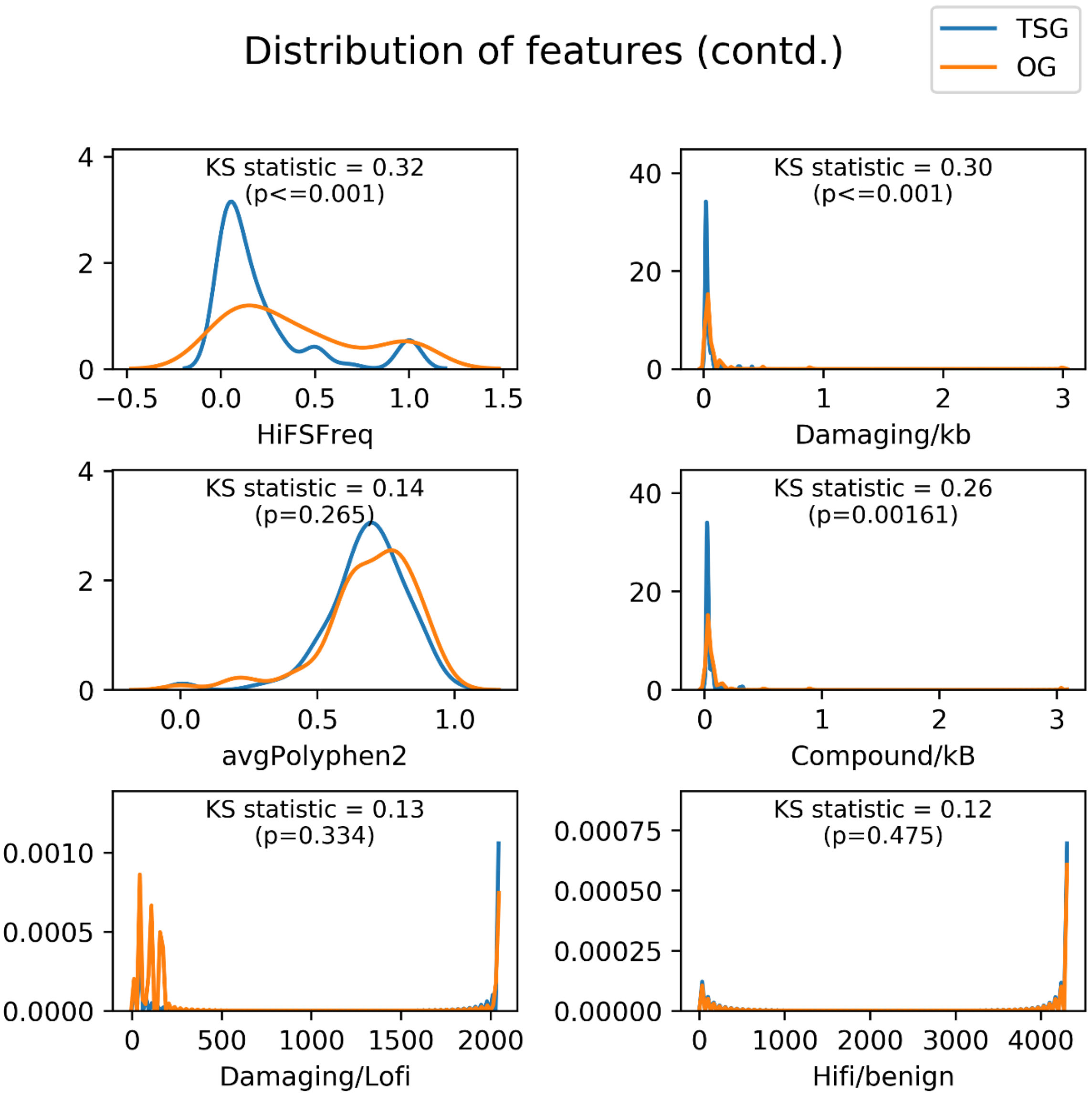

Supplementary Figure 1b Distribution of features used by the classifier for TSG and OG.

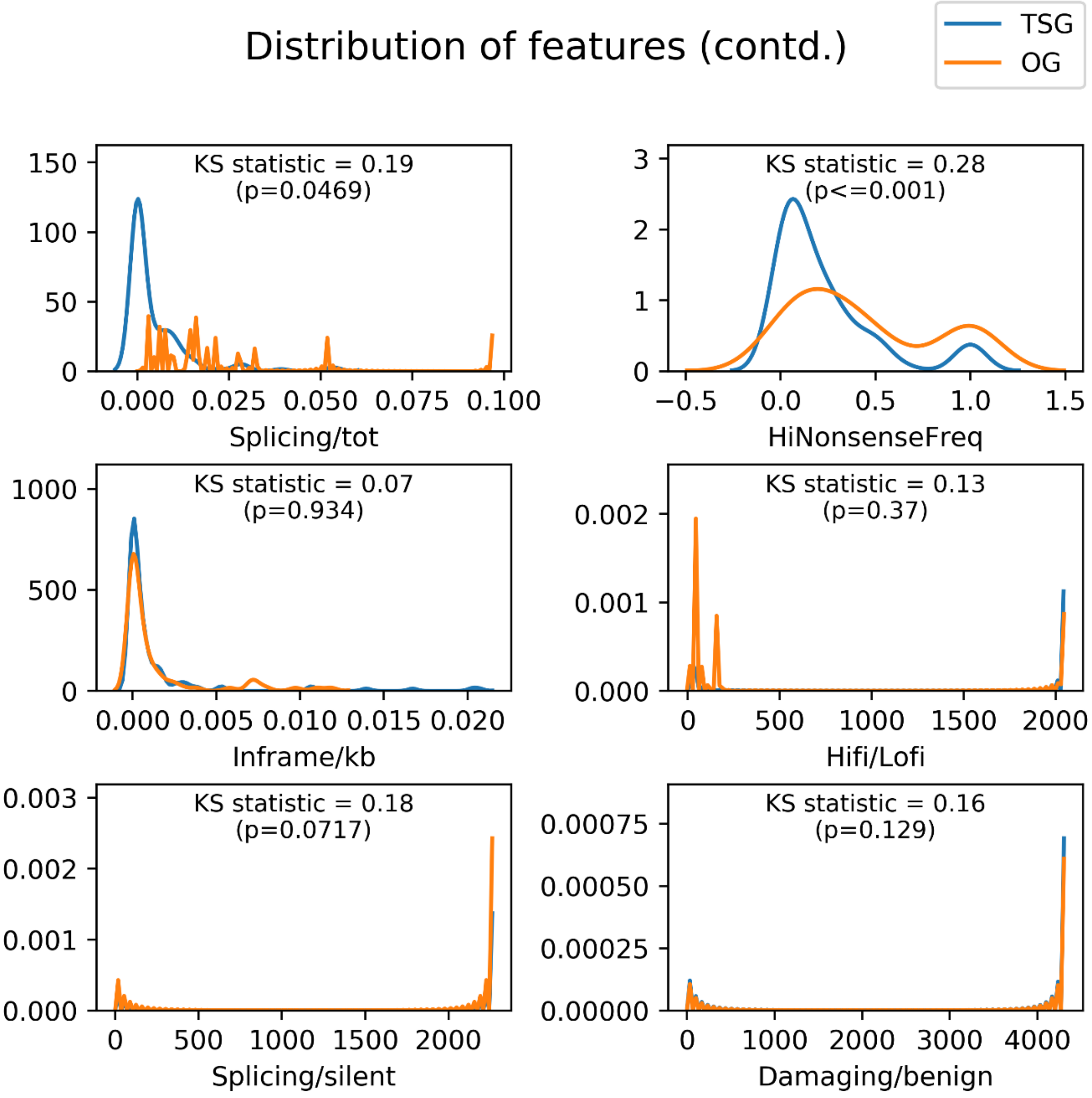

Supplementary Figure 1c Distribution of features used by the classifier for TSG and OG.

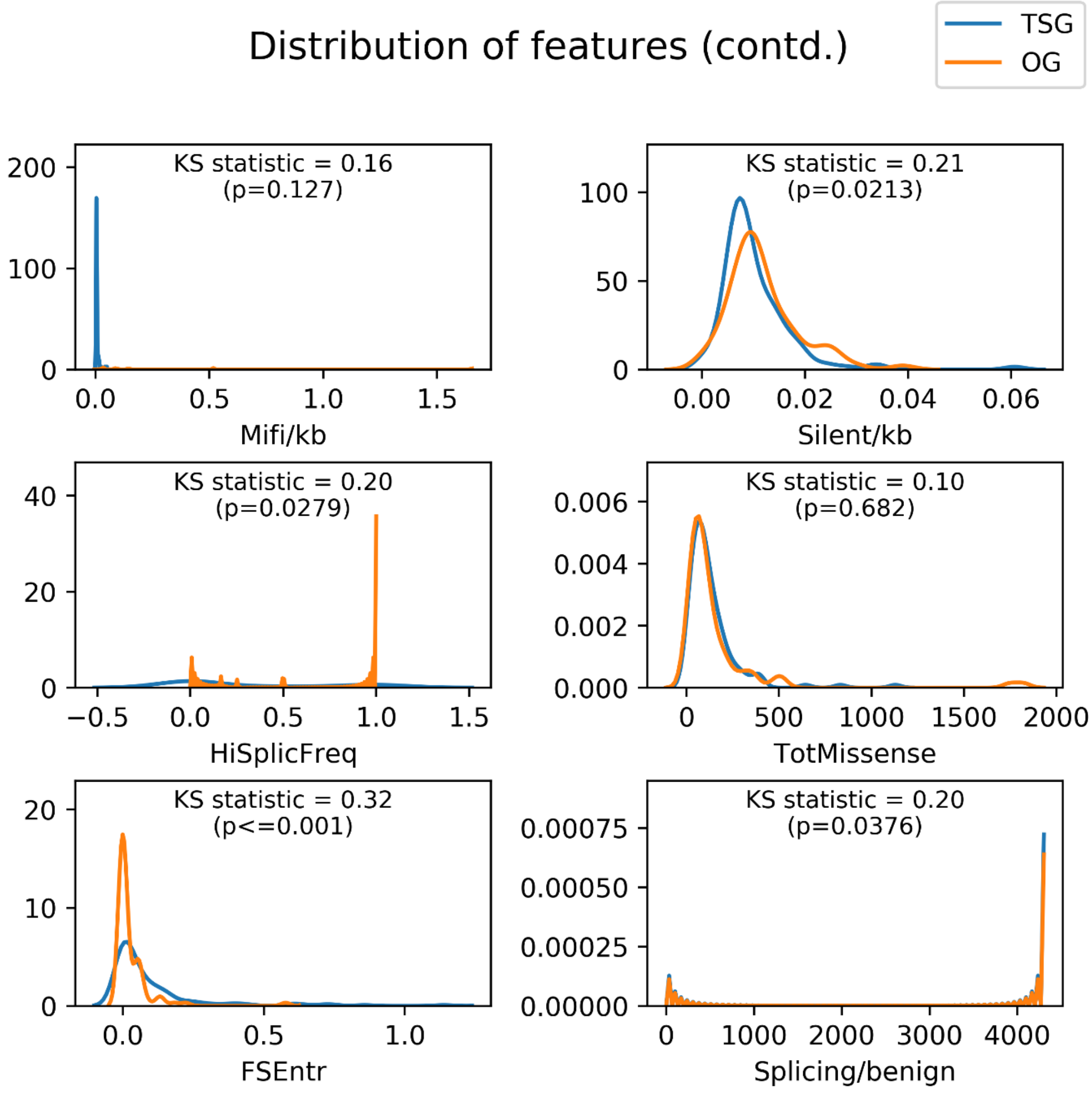

Supplementary Figure 1d Distribution of features used by the classifier for TSG and OG.

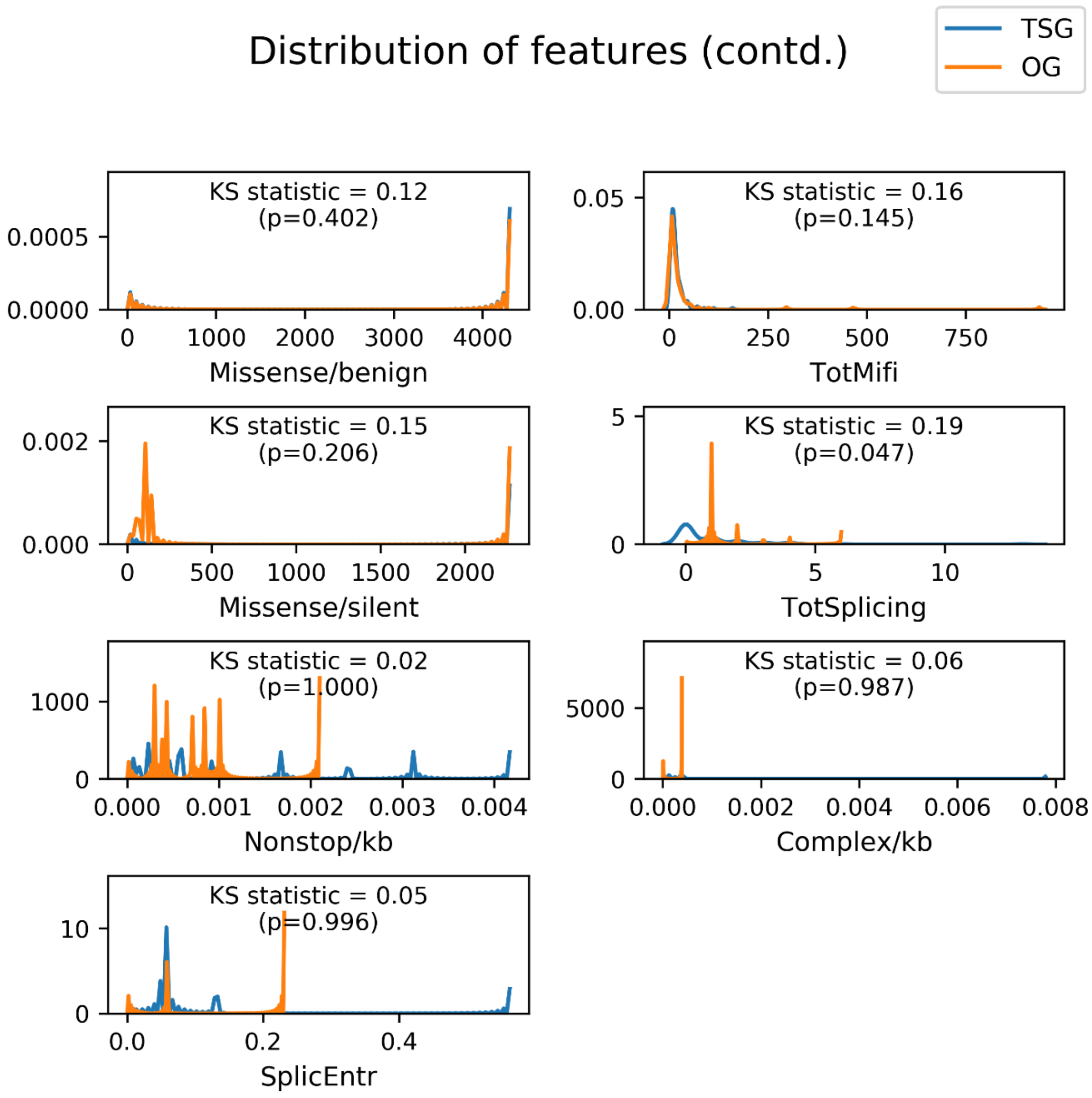

Supplementary Figure 1e Distribution of features used by the classifier for TSG and OG.

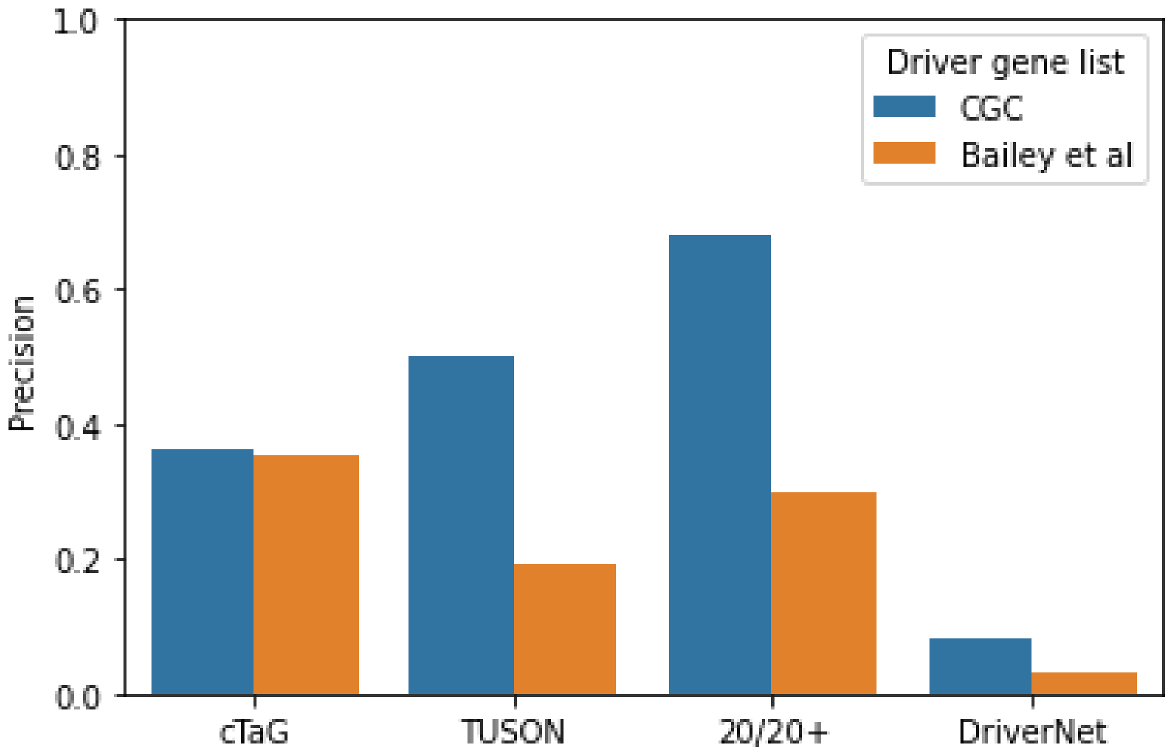

Supplementary Figure 2 Precision of various driver prediction tools. The list of known driver genes in CGC and genes published by Bailey et al were compared with the genes predicted by various computational driver prediction tools. The precision is defined as the number of known driver genes predicted by the total number of genes predicted.
